# Colorectal submucosal dissection using a novel traction method with a threaded clip attached to the outside of the lesion

**DOI:** 10.1055/a-2244-4428

**Published:** 2024-02-07

**Authors:** Keisaku Yamada, Masahiro Tajika, Tsutomu Tanaka, Nobuhito Ito, Akihiro Takagi, Yasumasa Niwa

**Affiliations:** 1 538363 Department of Endoscopy, Aichi Cancer Center Hospital, Nagoya, Japan

Colorectal endoscopic submucosal dissection (ESD) continues to present technical challenges. However, several reports indicate that traction devices can effectively reduce procedure times and complications associated with colorectal ESD 1 2 .

We developed a novel traction technique using a threaded clip attached to the outside of the lesion for lesions present in the greater curvature of the gastric body face-on to the scope 3 ; we termed this the “outside-the-lesion clip–thread method” (O-CTM). The advantage of this technique is that traction by the thread changes the angle of the lesion, making it align parallel with the scope rather than face-on to it, thus allowing ESD to be performed safely. In the case reported here, we applied this technique to a colorectal neoplasm in the cecum.

A 75-year-old man presented with a 30-mm 0-IIa lesion in the cecum ( Fig. 1 ) and underwent ESD ( Video 1 ). The lesion faced directly at the scope and thus was difficult to incise. For this reason, we decided to perform ESD using O-CTM with a balloon overtube (ST-CB1; Olympus Corporation).

**Fig. 1:**
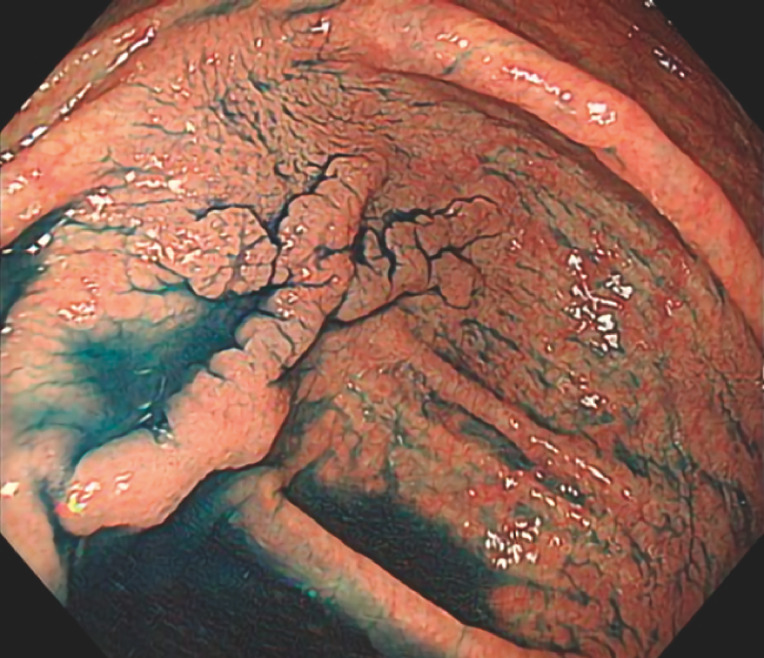
A 30-mm 0-IIa lesion in the cecum, face-on to the scope.

Colorectal endoscopic submucosal dissection using the outside-the-lesion clip–thread method enabled safe dissection of a colorectal adenoma in cecum.Video 1

Initially, a full circumferential incision was made. After that, the mucosa outside the full circumferential incision was grasped with grasping forceps and pulled to confirm the actual field of view, and the scope was pulled out from the balloon overtube that had already been inserted. A clip with a thread was attached to the scope, which was then reinserted. This clip was then attached to a pre-identified point, and traction on the thread changed the angle of the lesion, bringing it parallel to the scope ( Fig. 2 ). The submucosal dissection could then be performed safely, and en bloc resection was possible without complication. Pathological analysis revealed that the lesion was a severe adenoma with a negative margin ( Fig. 3 ).

**Fig. 2:**
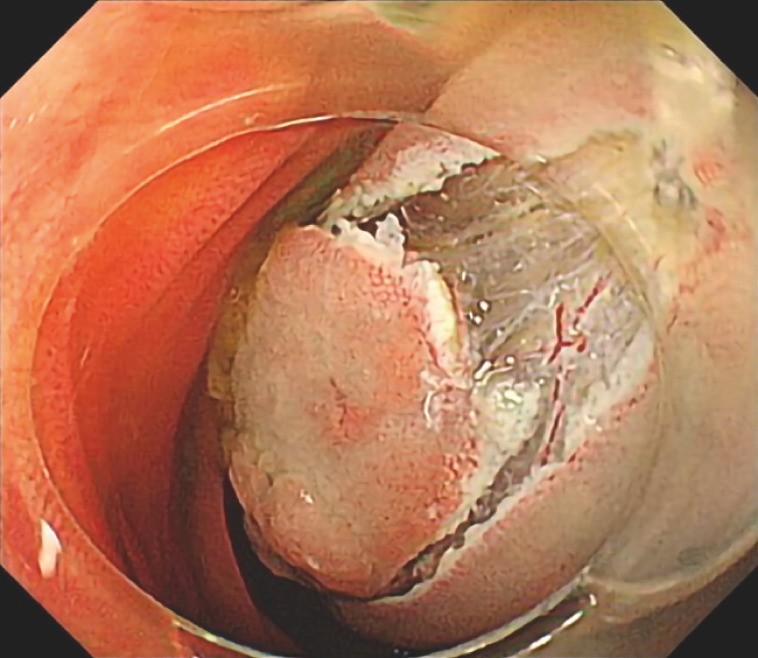
The face-on lesion was brought parallel to the scope by means of traction on the thread.

**Fig. 3:**
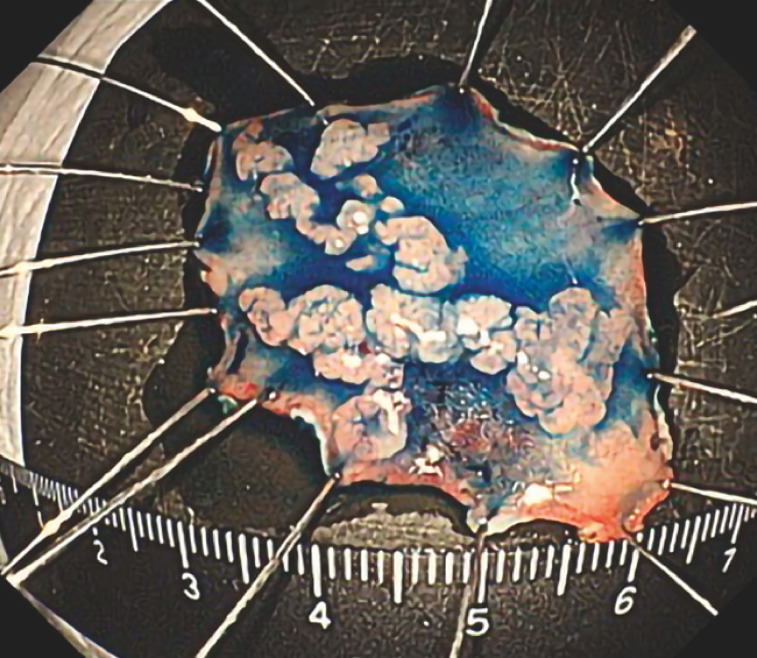
Histopathological section of the lesion showing an adenoma with negative margins.

Endoscopy_UCTN_Code_CPL_1AJ_2AD

## Notes

Endoscopy E-Videos https://eref.thieme.de/e-videos
*E-Videos* is an open access online section of the journal *Endoscopy* , reporting on interesting cases and new techniques in gastroenterological endoscopy. All papers include a high-quality video and are published with a Creative Commons CC-BY license. Endoscopy E-Videos qualify for HINARI discounts and waivers and eligibility is automatically checked during the submission process. We grant 100% waivers to articles whose corresponding authors are based in Group A countries and 50% waivers to those who are based in Group B countries as classified by Research4Life (see: https://www.research4life.org/access/eligibility/ ). This section has its own submission website at https://mc.manuscriptcentral.com/e-videos .
